# Supplementary figures and images for: A structural classification of the variant surface glycoproteins of the African trypanosome
Source: PLoS Negl Trop Dis. 2023 Sep 1;17(9):e0011621. doi: 10.1371/journal.pntd.0011621 (PMC10501684; doi:10.1371/journal.pntd.0011621)

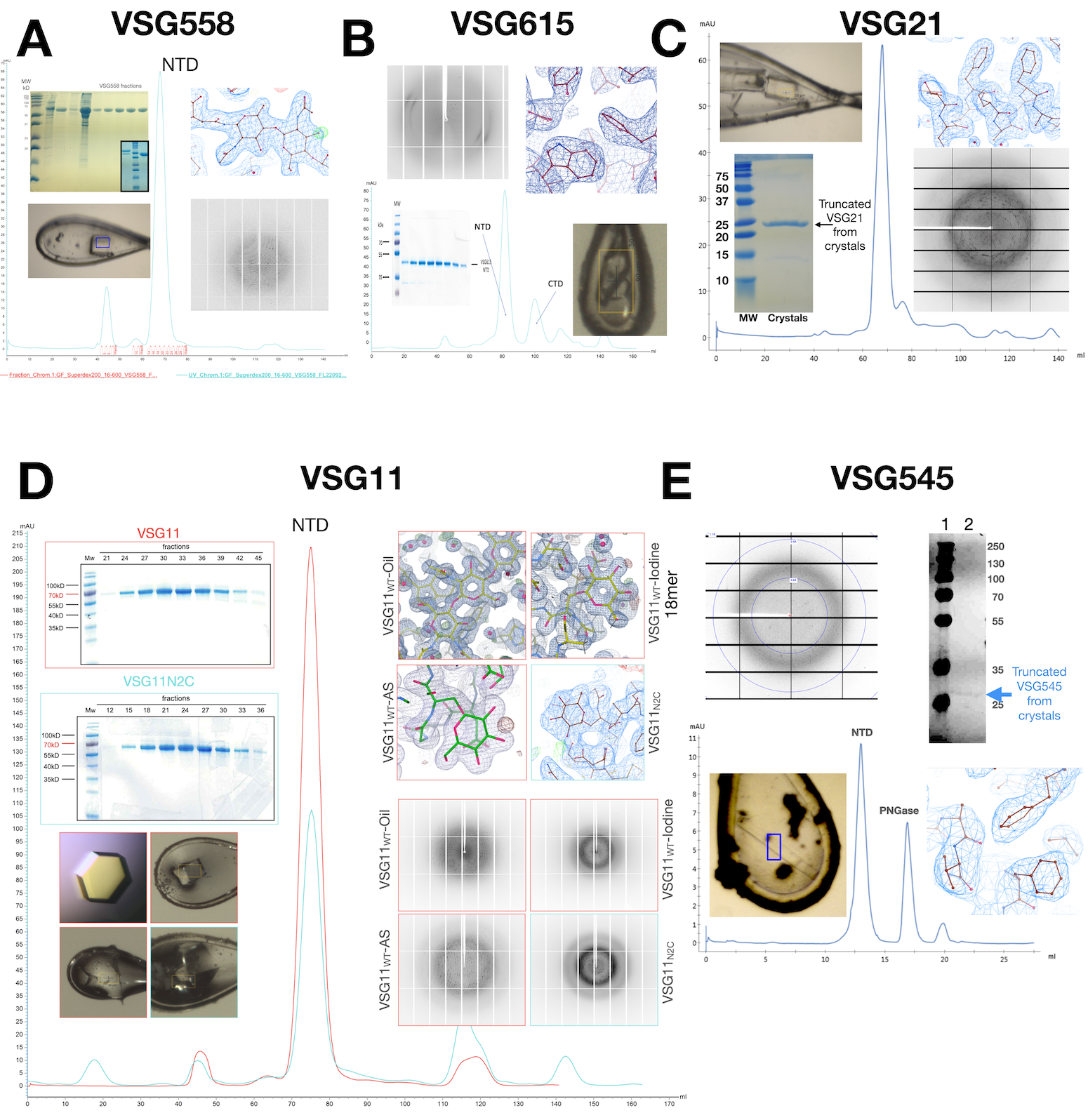

Supplement: S1 Fig — Summary of various steps in the crystallographic structural solution. Panels showing the gel filtration chromatogram (Superdex 200, Methods) of purified (A) VSG558, (B) VSG615, (C) VSG21 (D) VSG11 and (E) VSG545. A Coomassie stained SDS-PAGE gel of the final material used for crystallization, is shown, except for VSG21 and VSG545 which show an SDS-PAGE gel stained with Coomassie Blue of crystals of each VSG (crystals dissolved and run on the gel). The first lane shows molecular weight markers and the second the ~25kD band of the truncated protein in the crystals (highlighted with an arrow). Images of crystals grown in hanging drops, X-ray diffraction, and the final model 2Fo-Fc electron density contoured at 1σ are added. (TIFF) [file pntd.0011621.s001.tiff]

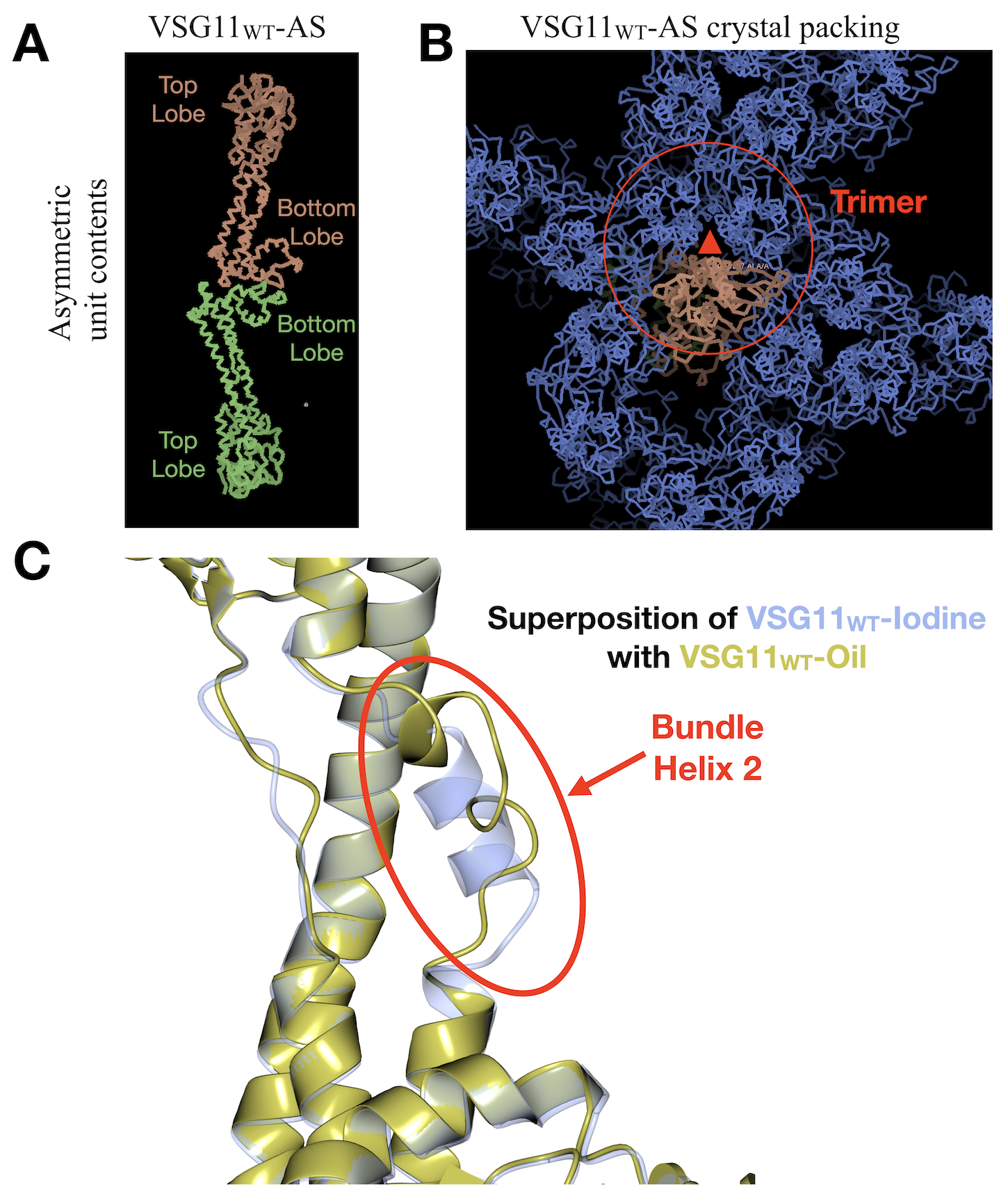

Supplement: S2 Fig — (A) The asymmetric unit of the wild type VSG11 NTD structure from ammonium sulfate. Two molecules of VSG11 are packed “head-to-tail” and shown in green and salmon. (B) The full crystal packing in the form from (A) with the salmon monomer shown and the crystal-packing that produces the standard B class trimer highlighted with a red circle. (C) A structural alignment of two wild type VSG11 models showing the conformation change at bundle helix 2 (VSG11WT-Iodine in blue, VSG11WT-Oil in gold). (TIFF) [file pntd.0011621.s002.tiff]

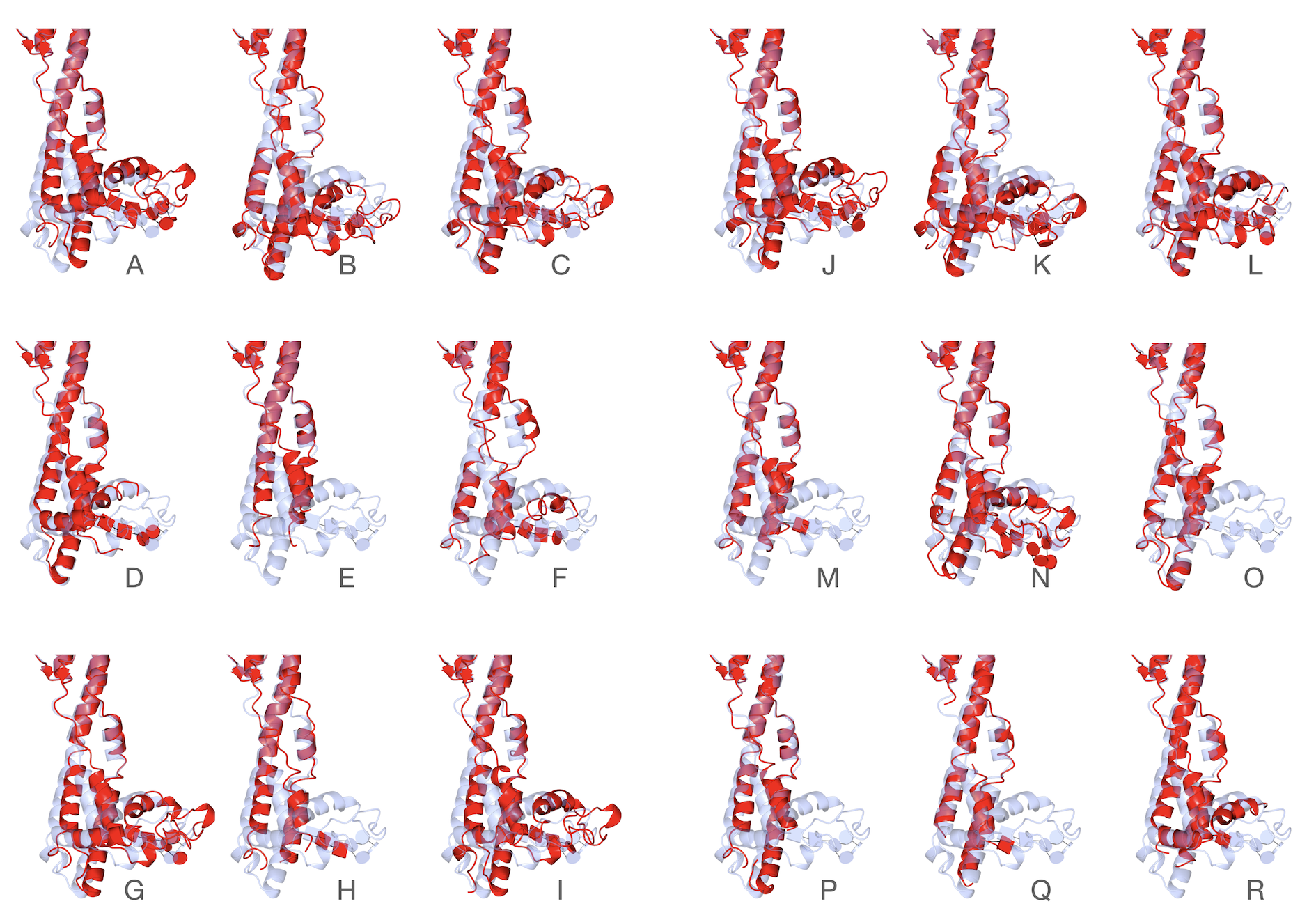

Supplement: S3 Fig — (A)-(R) show the VSG11WT-Iodine in light blue and a different, individual VSG11N2C monomer in red. Due to the high flexibility of the bottom lobes in the 18mer crystals, not all amino acids in some monomers of the bottom lobe could be modeled. (TIFF) [file pntd.0011621.s003.tiff]

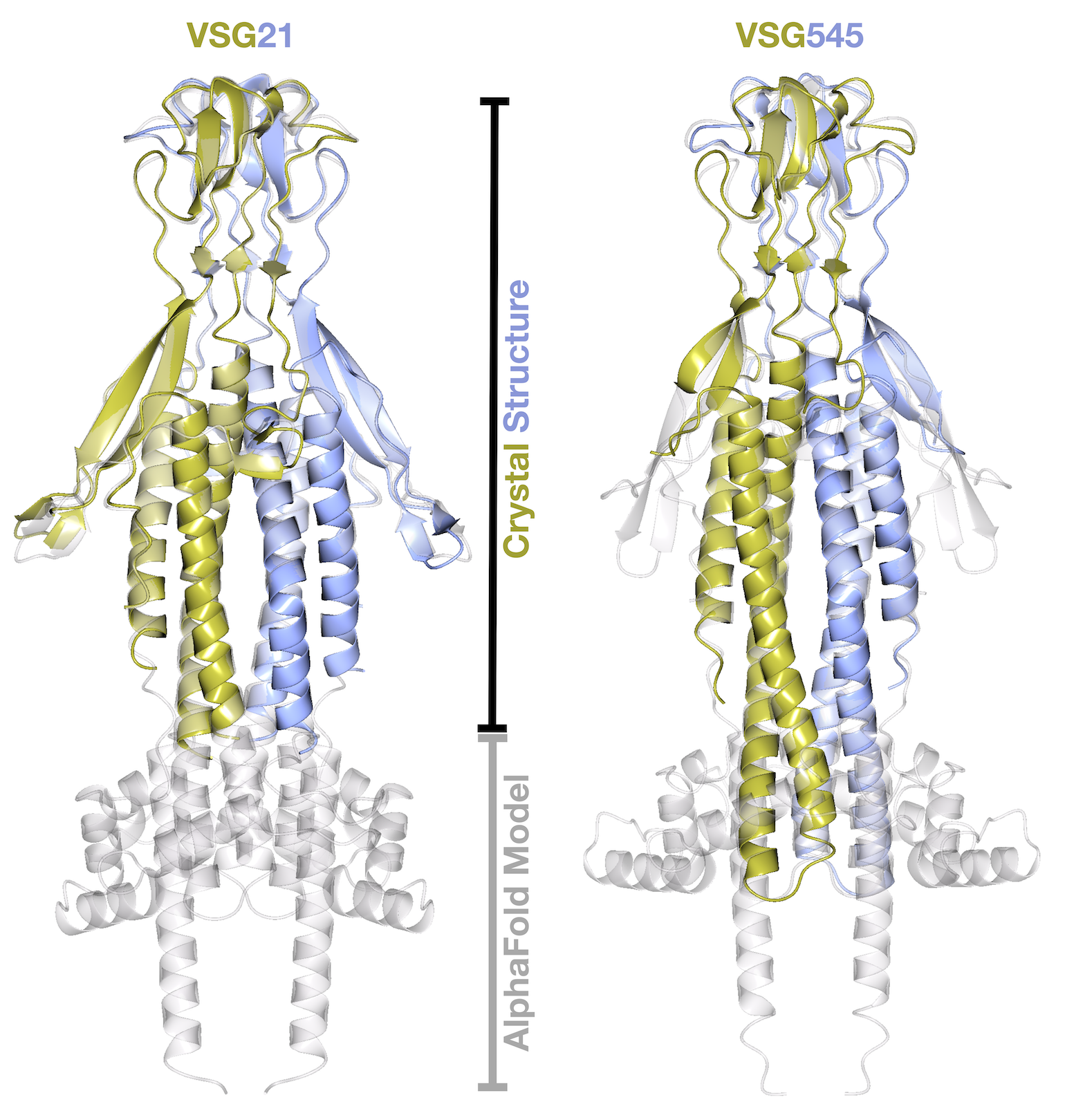

Supplement: S4 Fig — VSG21 (left) and VSG545 are colored in blue and gold (for the two chains in the dimer) and the respective AlphaFold models are light gray. (TIFF) [file pntd.0011621.s004.tiff]

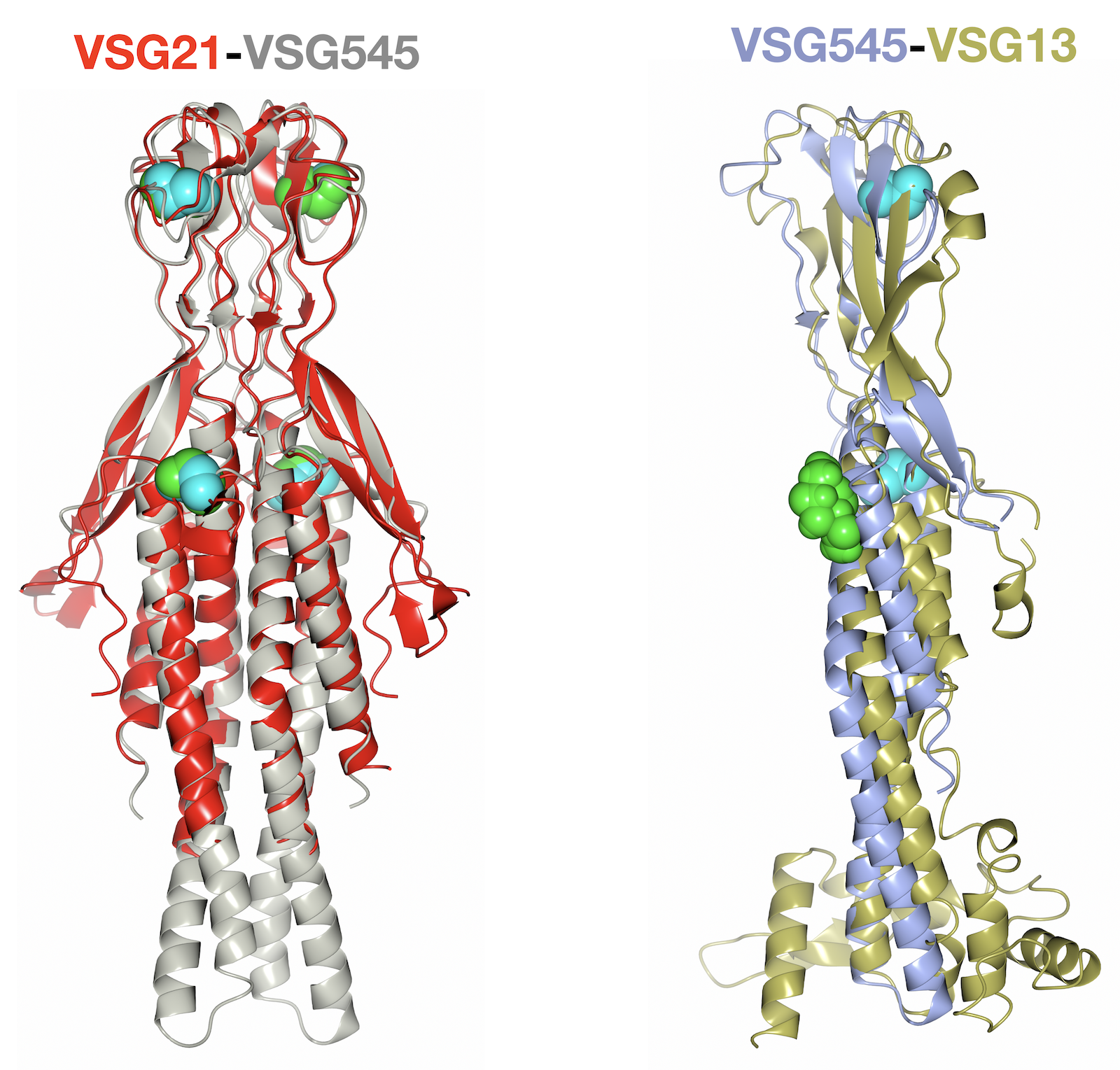

Supplement: S5 Fig — On the left an alignment of the A1c defining dimeric structures of VSG21 and VSG545 (red and gray, respectively) with disulfides in cyan (VSG21) and green (VSG545). On the right is a monomer alignment of A1c class member (VSG545 blue with cyan disulfides) with A1b class member VSG13 (gold with green disulfides). (TIFF) [file pntd.0011621.s005.tiff]

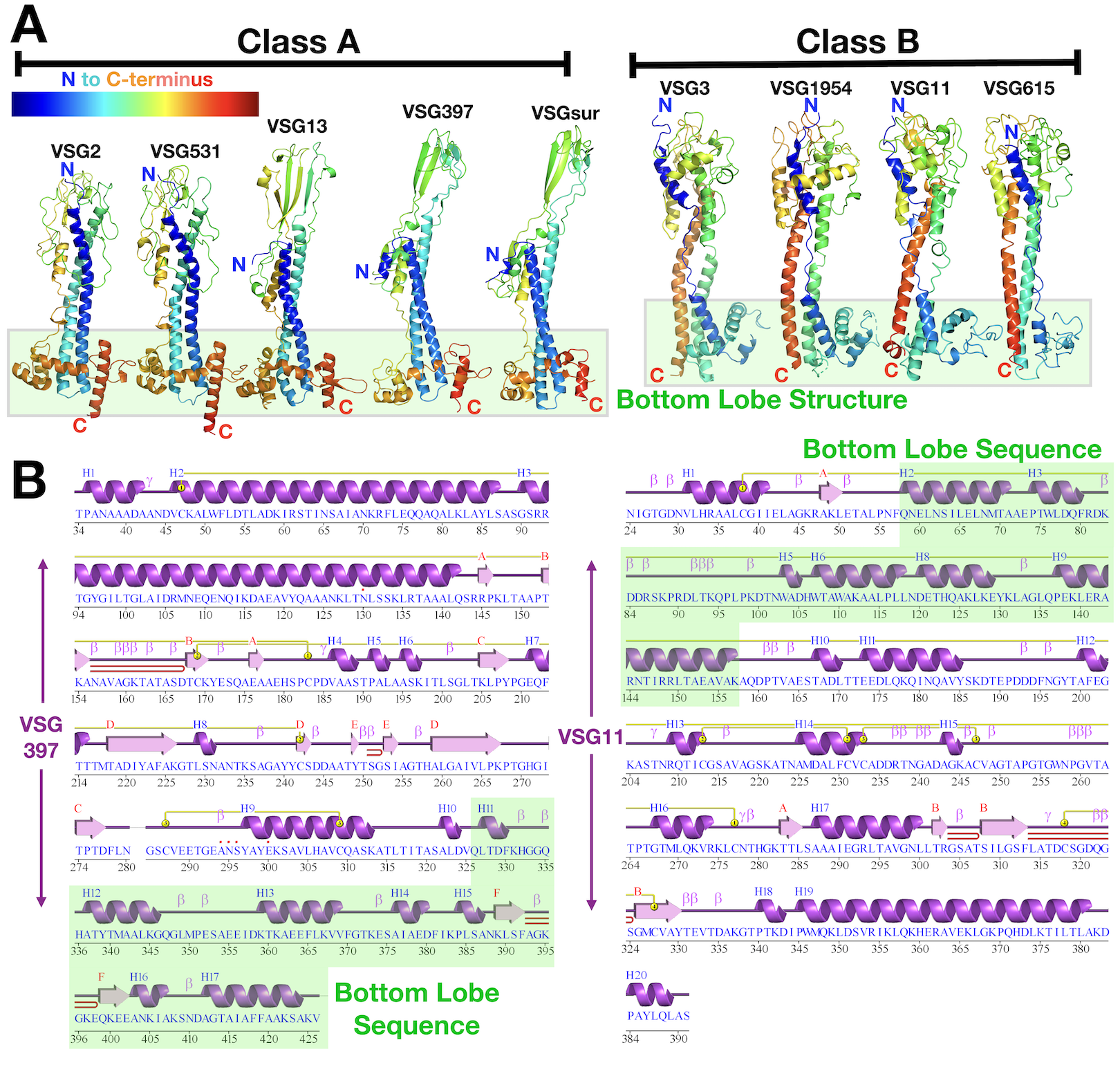

Supplement: S6 Fig — Topology of Class A and Class B (A) Two broad superfamily classes of VSGs identified through sequence analysis and defined by structural topology are shown here with representative structures. VSG monomers are shown as ribbon diagrams colored in a gradient from blue to red from N- to C-terminus. Structures reported in this manuscript are denoted in red and described in detail below. The lower, bottom lobe subdomains of the VSGs are highlighted in a green-tinted box. Structures drawn with CCP4mg. (B) Sequence and structurally-determined secondary structure of two representative VSGs from class A (VSG397) and class B (VSG11). The sequence region that forms the bottom lobe is indicated by green highlighting. Secondary structure illustrated with PDBSUM (https://www.ebi.ac.uk/thornton-srv/databases/pdbsum/). (TIFF) [file pntd.0011621.s006.tiff]

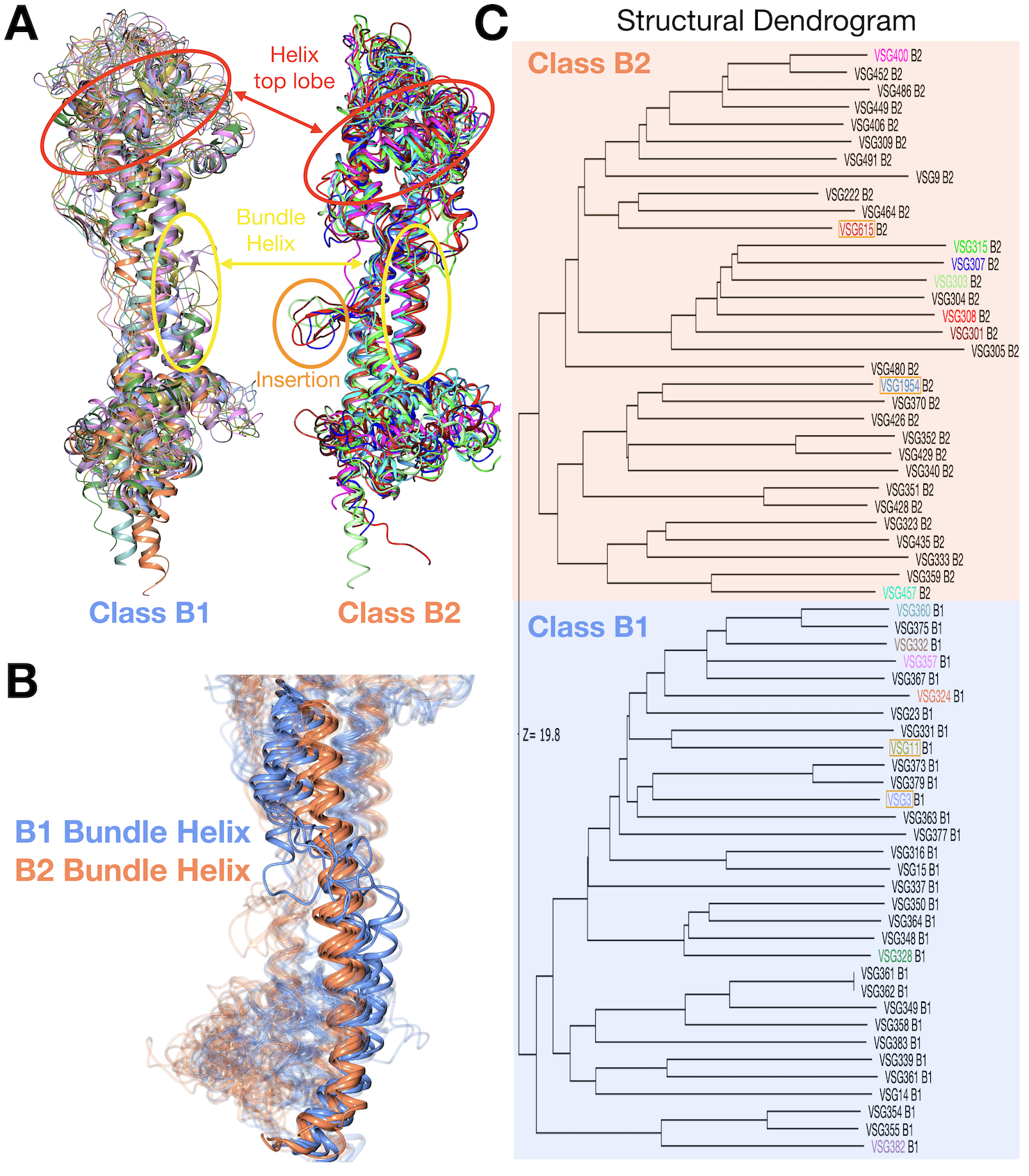

Supplement: S7 Fig — (A) Alignments of class B1 and B2 VSGs (experimental and AlphaFold models), highlighting the top lobe helices in B2 but not in B1 (red circle), the disordered bundle helix in B1 (yellow circle), and the inserted sequence in the bundle region (orange circle). The structural drawings match in color the names of the VSGs in panel (C). (B) Focus on the helixes in the 3-helix bundle differing between class B1 and B2. Orange is B2, showing that the helix is unbroken in most B2 VSGs. In B1 VSGs (light blue) the helix becomes disordered in the middle of the bundle and resumes a displaced helical structure afterward. (C) Structural dendrogram generated by the Dali Server comparing a number of experimental (VSG names with orange outline box) and AlphaFold models of the class B VSGs, showing that they split into two groups. B1 and B2 subgroups are shown with light blue and light orange backgrounds, respectively. (TIFF) [file pntd.0011621.s007.tiff]

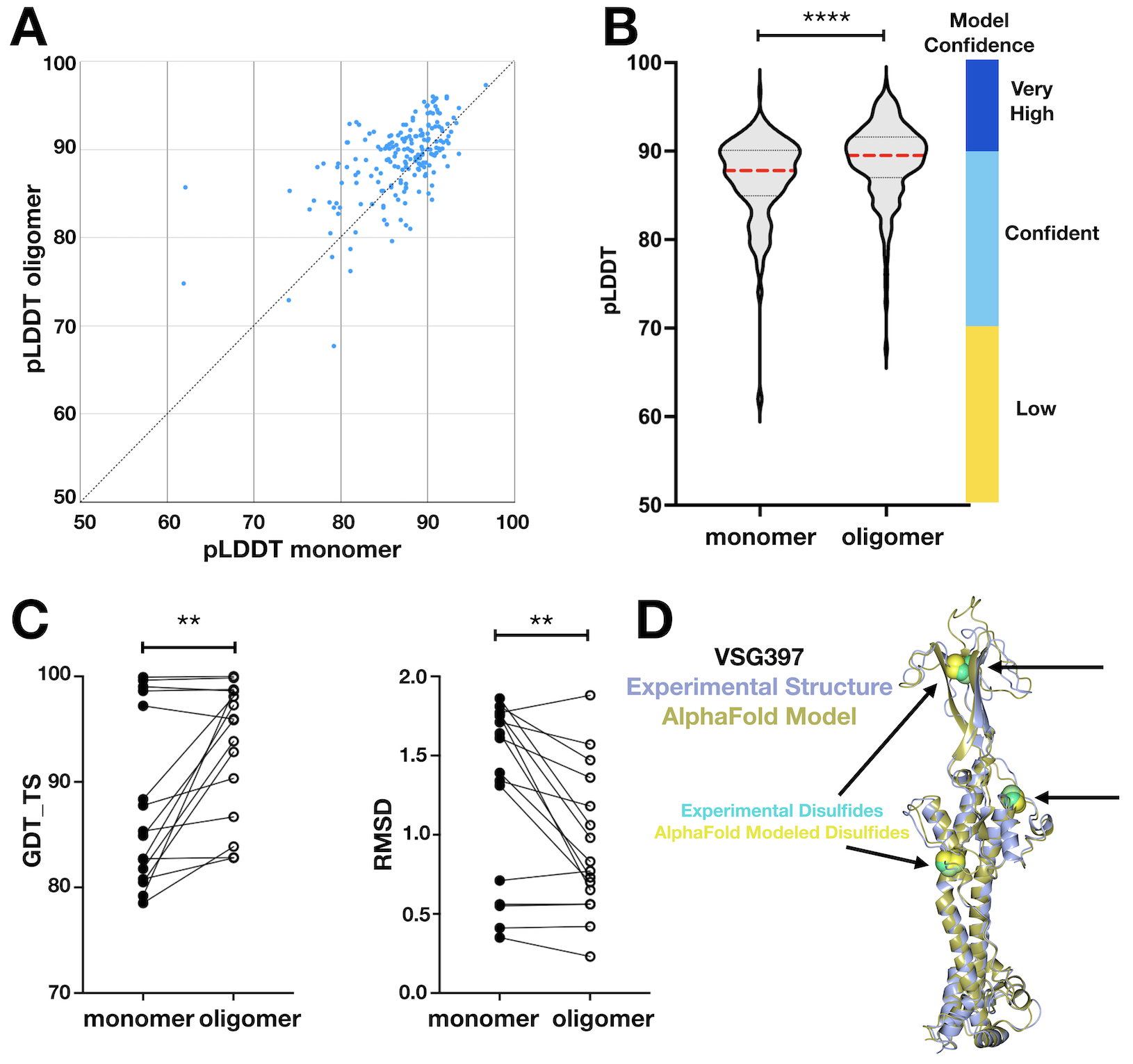

Supplement: S8 Fig — (A) Comparison of the predicted local distance test (pLDDT) for all predicted VSGs for the monomer compare to that for the oligomer. (B) Distribution of the predicted local distance test (pLDDT) for all predicted VSGs for the monomer and oligomer. The predicted local distance test (pLDDT) showed that the model confidence for all monomer predictions was 86.87 (standard deviation 4.831) and for the oligomer predictions 88.86 (standard deviation 4.438), evincing a statistically significant improvement in the pLDDT for the oligomer (mean difference of 1.992, P value <0.0001). (C) Comparison of the X-ray structures with the corresponding predictions (monomers and oligomers) showed a higher GDT_TS for the oligomer 93.52 (standard deviation 6.257) compared to the monomer 87.94 (standard deviation 8.101) with a mean of difference 5.583, P value 0.004, and a lower RMSD for the oligomer 0.9344 Å (standard deviation 0.4547 Å) compared to the monomer 1.283 Å (standard deviation 0.562 Å) with a mean of difference -0.3488 Å, P value 0.0027. (D) Comparison of experimental and AlphaFold predicted structures of VSG397. Ribbon diagrams of the VSG397 monomers are shown in light blue and gold for the experimental and predicted structures, respectively. Disulfides are shown in cyan and yellow for the experimental and predicted structures, respectively, marked by black arrows. For P values, P > 0.05 is considered not significant, "*" indicates a P ≤ 0.05, "**" indicates a P ≤ 0.01, "***" indicates P ≤ 0.001, and "****" indicates a P ≤ 0.0001. (TIFF) [file pntd.0011621.s008.tiff]

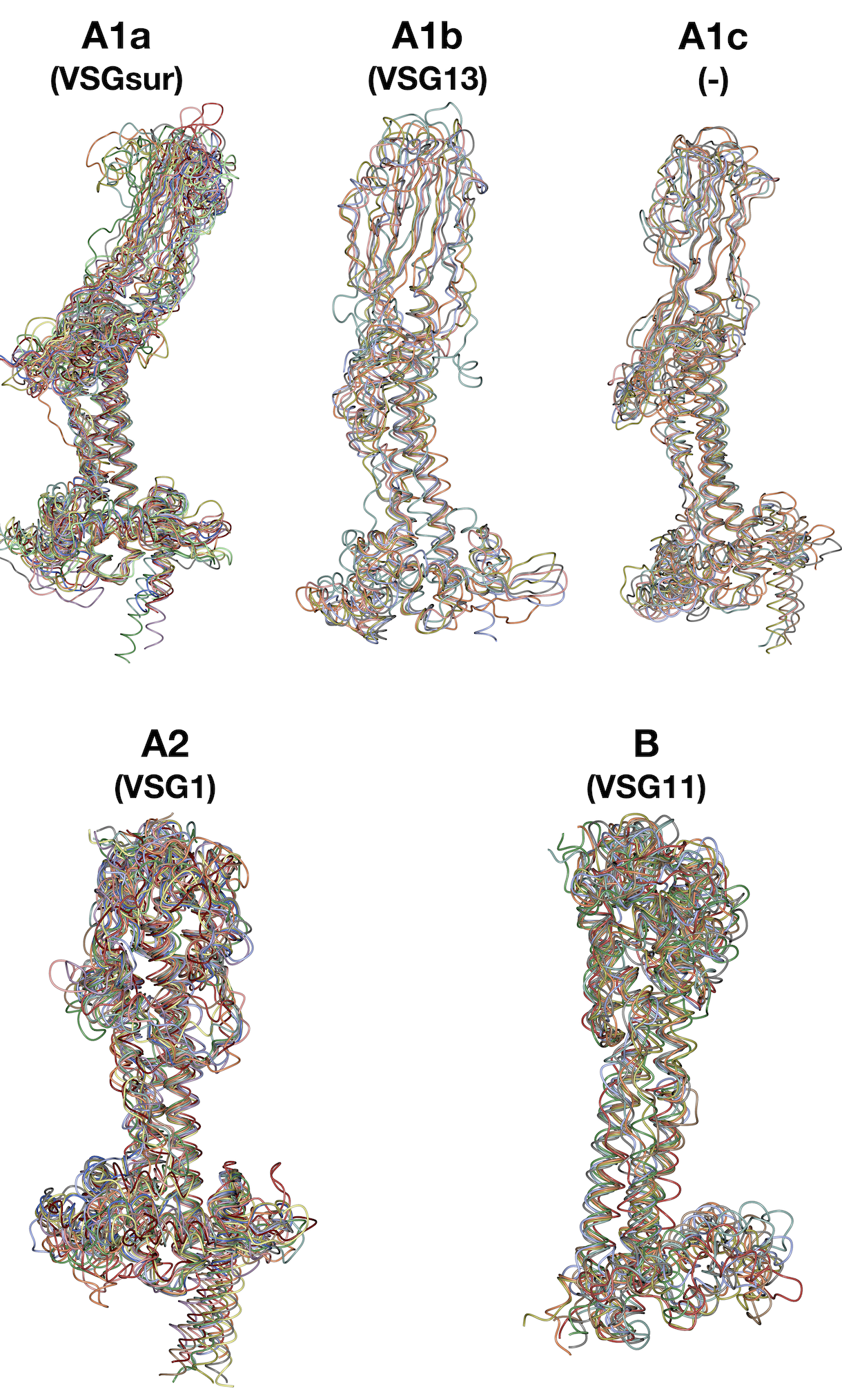

Supplement: S9 Fig — Structures of aligned VSG monomers are drawn in different colors with a thin worm style. (A) Class A1a with models for the UniProtKB/Swiss-Prot accession numbers Q580P4, Q580P5, Q57TR8, Q57TR3, Q38CQ8, Q57X40, Q583L8, Q583L5, Q57X41, Q22KU2, Q38G13, Q380W0, and Q38CP5 aligned to VSGsur. (B) Class A1b with models for the UniProtKB/Swiss-Prot accession numbers Q57Y76, Q57X39, Q4GY52, Q586M4, Q57XI7 aligned to VSG13. (C) Class A1c with models for the UniProtKB/Swiss-Prot accession numbers Q380U5, Q380V8, Q380U9, Q380Y0, Q580N8, Q38CP2 aligned to an AlphaFold model for VSG21. (D) Class A2 with models for the UniProtKB/Swiss-Prot accession numbers Q57X38, Q580N9, Q57Z50, Q4FKU3, Q380X8, Q57XH3, Q38G16, Q57TR9, Q380W1, Q38CQ1, Q583L4, Q38G20 aligned to VSG1. (E) Class B with models for the UniProtKB/Swiss-Prot accession numbers Q380U8, Q583L3, Q4FKE9, Q38CP0, Q57TR6, Q387P0, Q57TR7, Q4GY50 aligned to VSG11. Alignments generated and illustrated with CCP4mg, each structure aligned in a different color. (TIFF) [file pntd.0011621.s009.tiff]

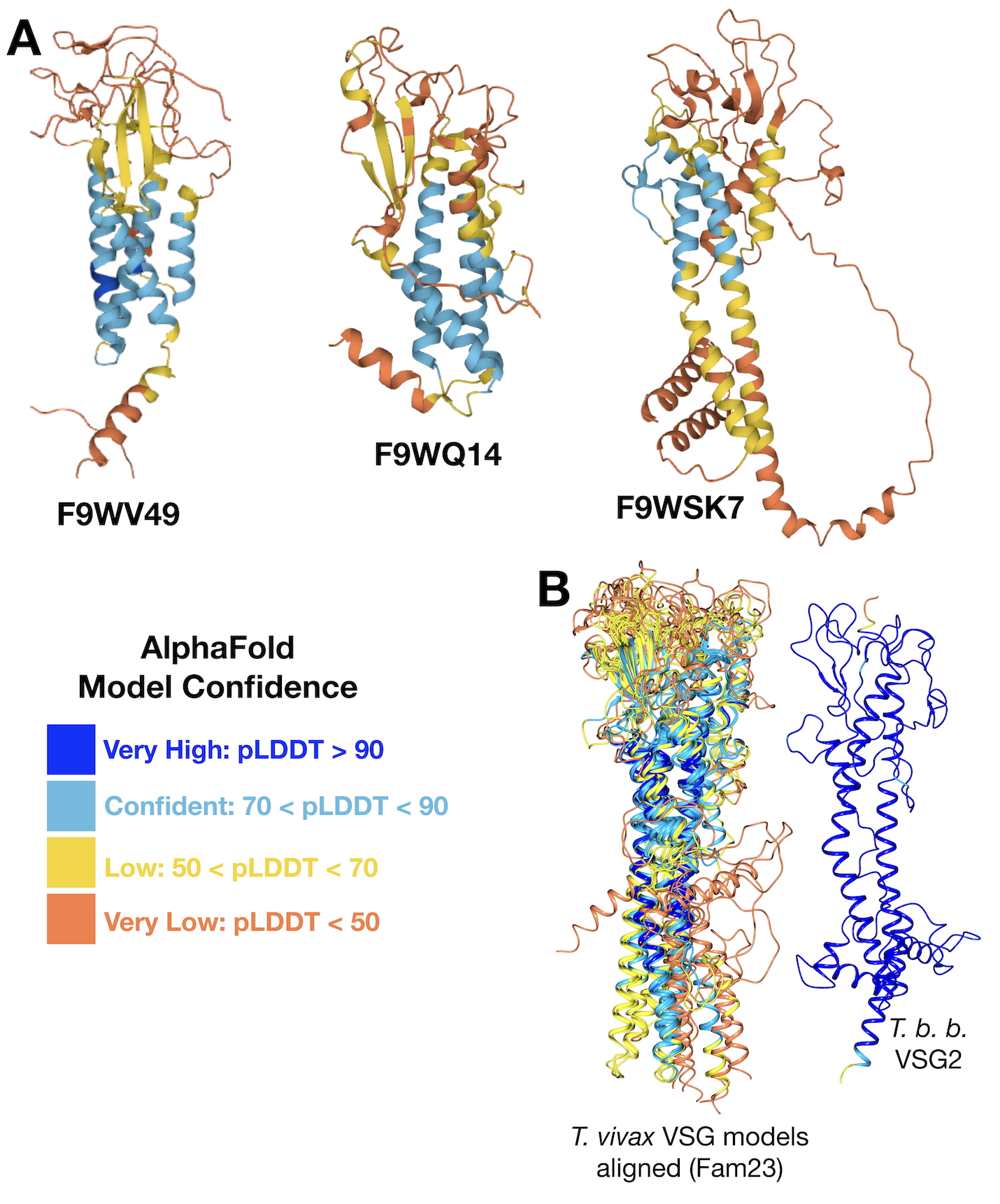

Supplement: S10 Fig — (A) A collection of several T. vivax VSG protein structures predicted by AlphaFold and colored by pLDDT confidence score (as indicated). (B) Comparison of several T. vivax putative VSG structures (from Fam23 of the Fam23-26 VSG gene groups) and the prediction of T. brucei brucei VSG2, both colored by pLDDT confidence scores. (TIFF) [file pntd.0011621.s010.tiff]

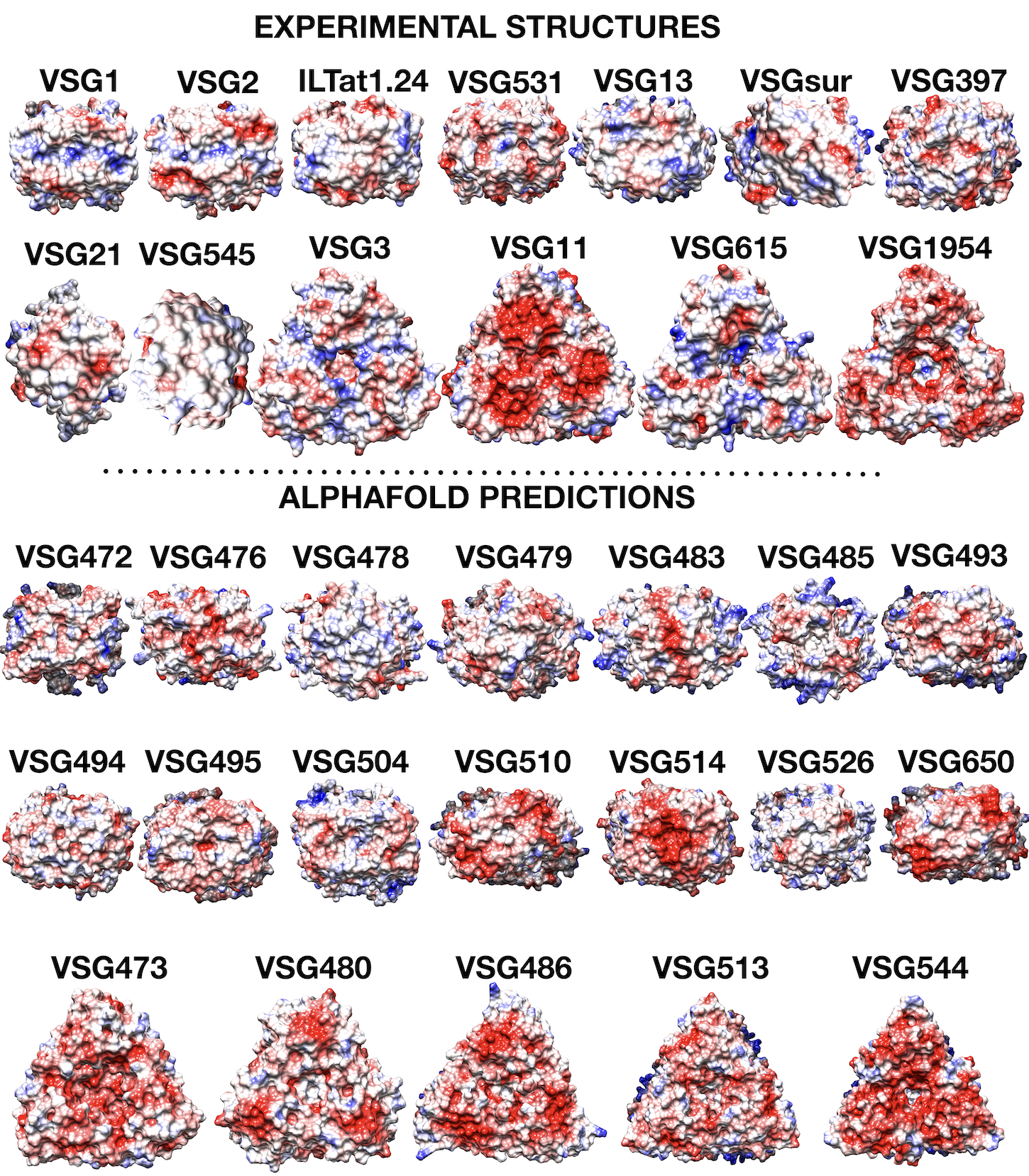

Supplement: S11 Fig — Colored by charge distribution with blue indicating positive or basic, white neutral, and red acidic or negative (produced with Chimera using the “Coulombic Surface Coloring” option). The top portion shows surfaces from several experimental structures, the bottom the surfaces from models produced by AlphaFold (in either the dimeric or trimeric assemblies). (TIFF) [file pntd.0011621.s011.tiff]
