# Supplementary material for: A structural classification of the variant surface glycoproteins of the African trypanosome
Source: PLoS Negl Trop Dis. 2023 Sep 1;17(9):e0011621. doi: 10.1371/journal.pntd.0011621 (PMC10501684; doi:10.1371/journal.pntd.0011621)
Supplement: S1 Table — (PDF) [file pntd.0011621.s012.pdf]

Table S1: Crystallographic Statistics

Parameter	VSG11$_{WT}$-Iodine	VSG11$_{WT}$-Oil	VSG11$_{WT}$-AS	VSG11$_{N2C}$-18mer	VSG615	VSG558	VSG21	VSG545
Wavelength	1.00 Å	1.00 Å	1.00 Å	1.00 Å	1.00 Å	1.00 Å	1.00Å	1.00Å
Resolution range	41.26-1.27 (1.32-1.27)	35.28-1.23 (1.27-1.23)	42.45-1.75 (1.81-1.75)	48.81-2.59 (2.68-2.59)	68.16-3.22 (3.34-3.22)	53.50-1.74 (1.80-1.74)	47.28 -2.01 (2.08 -2.01)	44.41-3.15 (3.26 - 3.15)
Space group	P 3 2 1	P 3 2 1	P 3 2 1	P 1 21 1	P 1 21 1	P 1 21 1	C 1 2 1	P 21 21 21
Unit cell	75.43 75.43 106.4 90 90 120	74.86 74.86 105.61 90 90 120	76.24 76.24 212.26 90 90 120	131.9 210.77 133.75 90 104.54 90	86.28 111.23 108.59 90 91.65 90	103.08 64.12 155.04 90 109.13 90	174.329 84.422 120.095 90 100.89 90	46.41 52.28 305.80 90 90 90
Total reflections	901512 (86366)	938886 (70182)	1384124 (124473)	1484781 (140952)	186820 (20643)	1170852 (109790)	780507 (72645)	64987 (6184)
Unique reflections	92763 (9185)	99721 (9827)	72926 (7093)	217669 (20848)	30929 (3271)	196293 (19469)	111483 (10775)	13575 (619)
Multiplicity	9.7 (9.4)	9.4 (7.1)	19.0 (17.5)	6.8 (6.8)	6.0 (6.3)	6.0 (5.6)	7.0 (6.7)	4.8 (4.6)
Completeness (%)	99.96 (100)	99.91 (99.75)	99.75 (97.77)	99.42 (95.33)	92.38 (97.84)	98.72 (93.25)	97.85 (95.61)	75.58 (45.29)
Mean I/sigma(I)	14.54 (1.27)	15.79 (0.98)	19.09 (0.98)	14.56 (1.22)	3.71 (0.63)	5.36 (0.39)	8.17 (1.19)	5.63 (1.18)
Wilson B-factor	14.58	13.37	32.69	66.92	85.19	30.85	38.05	36.44
R-merge	0.0903 (1.683)	0.0754 (1.777)	0.09623 (2.514)	0.09065 (1.294)	0.3959 (2.754)	0.1614 (3.64)	0.1658 (2.154)	0.2451 (1.656)
R-meas	0.0954 (1.781)	0.0796 (1.918)	0.09892 (2.588)	0.0983 (1.402)	0.4333 (2.998)	0.1765 (4.017)	0.1793 (2.336)	0.2753 (1.872)
R-pim	0.0304 (0.5777)	0.0258 (0.7099)	0.02264(0.6037)	0.03766(0.5325)	0.1736(1.171)	0.07051 (1.671)	0.06764 (0.8953)	0.122 (0.8506)
CC1/2	0.999 (0.471)	0.999 (0.388)	1 (0.348)	0.999 (0.545)	0.978 (0.127)	0.996 (0.268)	0.996 (0.53)	0.993 (0.785)
CC*	1 (0.8)	1 (0.748)	1 (0.718)	1 (0.84)	0.995 (0.474)	0.999 (0.65)	0.999 (0.832)	0.998 (0.938)
Reflections (refinement)	92761 (9185)	99650 (9807)	72898 (7073)	217644 (20848)	30847 (3257)	193856 (18159)	111437 (10769)	10352 (616)
Reflections (R-free)	4639 (460)	4979 (489)	3645 (355)	10882 (1043)	1524 (171)	9780 (822)	5573 (539)	535 (31)
R-work	0.2063 (0.2937)	0.1614 (0.2734)	0.1991 (0.3171)	0.2292 (0.3530)	0.3070 (0.3828)	0.2378 (0.5083)	0.1797 (0.3159)	0.2359 (0.3296)
R-free	0.2300 (0.29927)	0.1863 (0.3119)	0.2378 (0.3486)	0.2696 (0.3642)	0.3245 (0.4016)	0.2610 (0.5356)	0.2187 (0.3487)	0.2886 (0.3648)
CC(work)	0.943 (0.692)	0.964 (0.657)	0.957 (0.609)	0.932 (0.632)	0.891 (0.279)	0.957 (0.601)	0.965 (0.785)	0.763 (0.405)
CC(free)	0.934 (0.680)	0.960 (0.503)	0.957 (0.777)	0.915 (0.579)	0.889 (0.201)	0.946 (0.534)	0.935 (0.688)	0.876 (0.275)
all atoms*	3338	3317	6295	43982	14118	12359	10970	3452
Protein atoms	2782	2822	5604	42850	13883	11033	10079	3452
ligands	175	154	240	848	222	565	0	0
solvent	452	412	451	284	13	761	891	0
Protein residues	366	368	736	5854	1887	1444	1398	483
RMS(bonds)	0.012	0.004	0.008	0.004	0.005	0.007	0.006	0.003
RMS(angles)	1.24	0.83	1.06	0.63	0.81	0.95	0.75	0.54
Ramachandran favored (%)	97.25	97.81	96.31	93.30	84.44	96.03	98.09	96.38
Ramachandran allowed (%)	2.75	2.19	3.55	6.26	14.63	3.69	1.91	3.62
Ramachandran outliers (%)	0.00	0.00	0.14	0.43	0.93	0.28	0.00	0.00
Rotamer outliers (%)	0.70	0.33	2.86	3.79	3.72	2.25	1.48	0.28
Clashscore	2.11	1.73	2.94	6.80	13.41	2.58	2.52	6.06
Average B	20.70	20.73	42.79	95.76	88.44	44.06	51.26	42.89
protein	19.77	19.21	42.38	95.64	88.42	43.88	51.46	42.89
ligands	21.35	24.55	50.80	114.45	91.57	49.56	N/A	N/A
solvent	26.29	30.43	43.61	59.16	20.56	42.58	48.91	42.89
TLS groups	6		15	111		24	29	12

Statistics for the highest-resolution shell are shown in parentheses. *All atoms refers to non-hydrogen atoms
